# Role of elective neck dissection in cT2N0 maxillary sinus squamous cell carcinoma

**DOI:** 10.1038/s41598-024-66179-7

**Published:** 2024-07-14

**Authors:** Jingjing Wang, Qigen Fang, Xu Zhang, Liyuan Dai, Ruihua Luo

**Affiliations:** Department of Head Neck and Thyroid, The Affiliated Cancer Hospital of Zhengzhou University & Henan Cancer Hospital, Zhengzhou, 450008 China

**Keywords:** Maxillary sinus squamous cell carcinoma, Elective neck dissection, Occult metastasis, Survival, PSM, Head and neck cancer, Oral cancer, Cancer, Surgical oncology

## Abstract

Our objective was to examine the impact of elective neck dissection (END) on the prognosis of patients with cT2N0 maxillary sinus squamous cell carcinoma (MS-SCC) and to determine factors that predict the occurrence of occult metastasis in this patient population. A retrospective analysis was conducted using data from the SEER database. Patients with cT2N0 MS-SCC were included in the study and divided into two groups: those who received END and those who did not. The impact of END on disease-specific survival (DSS) and overall survival (OS) was assessed using propensity score matching. Multivariate logistic regression analysis was performed to determine predictors for occult metastasis. A total of 180 patients were included in the study, with 40 cases receiving END. Following propensity score matching, patients treated with END and those without showed similar DSS and OS rates. Occult metastasis was observed in 9 patients, corresponding to a rate of 22.5%. High-grade tumors were independently associated with a higher risk of occult metastasis compared to low-grade tumors (hazard ratio 1.52, 95% confidence interval 1.17–2.00). cT2 MS-SCC carries an occult metastasis rate of 22.5%, with histologic grade being the primary determinant of occult metastasis. END does not confer a significant survival benefit in this patient population.

## Introduction

Maxillary sinus squamous cell carcinoma (MS-SCC) is an infrequent malignancy but represents the predominant type of cancer affecting the nasal cavity and paranasal sinus^1^, due to its early asymptomatic nature, over 80% of newly diagnosed cases of MS-SCC present at an advanced T3/4 stage. Curative treatment always includes surgery and radiotherapy with or without chemotherapy^2^. In contrast to squamous cell carcinoma occurring in other areas of the head and neck, MS-SCC typically exhibits a reduced propensity for lymph node (LN) metastasis^3^. NCCN guideline does not clearly describe how a cN0 neck should be managed^4^, several experts endorse the treatment of cN0 neck in cT3/4 MS-SCC^5–8^. However, it is not yet established whether active neck intervention confers a survival advantage for cT2 tumors.

It is widely accepted that elective neck dissection (END) should be performed when the risk of occult metastasis exceeds 15–20%^9^. Traditionally, observation alone has sufficed for neck management in cT1/2N0 MS-SCC due to a lack of lymphatic network. However, recent studies have reported a higher incidence of LN metastasis in cT2 MS-SCC with palate invasion, indicating a substantial potential for metastasis. In fact, this type of disease exhibits a similar frequency of occult metastasis to that seen in cT3/4 tumors^10,11^. Recent reviews also suggest that END is advisable for cT3/4 rather than cT1 MS-SCC, with special consideration given to cT2 tumors^1,12^.

Therefore, our objective was to investigate the impact of END on prognosis and to determine the frequency of occult metastasis, as well as the factors predicting its occurrence, in cT2N0 MS-SCC.

## Patients and methods

### Ethnic consideration

Ethical approval was not required for this study as the data are publicly accessible. The need for consent was waived by the ethics committee.

### Patient selection

**Figure 1:**
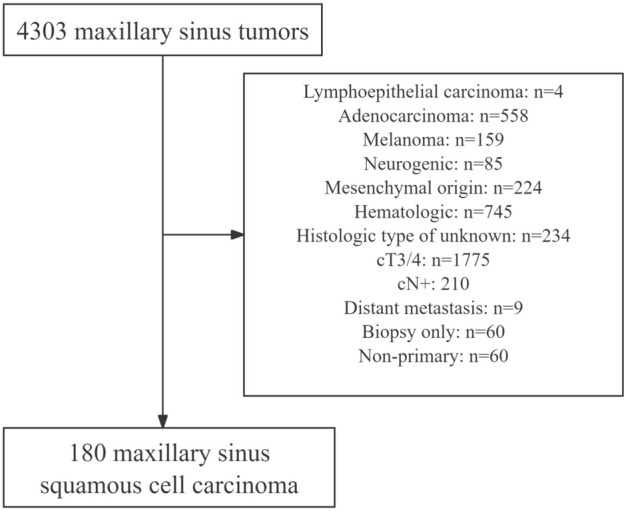
Flowchart of the 180 enrolled patients.

All the data was obtained from the SEER database [incidence-SEER research data, 17 registries, November 2022 Sub (2000–2020)] on January 1, 2024. The SEER database aims to provide vital information on cancer statistics and diminish the cancer burden among the population of the United States. Among the 4303 patients diagnosed with maxillary sinus tumor, only 180 met the inclusion criteria after excluding 4123 patients (Fig. 1). We collected relevant information on the patients' demographics, pathology, treatment, and follow-up.

### Variable definition

cN0 was defined as the absence of any clinically positive lymph nodes, based on the 6th, 7th, and 8th AJCC classification system. Tumor stage was classified according to the 6th, 7th, and 8th AJCC system. The histological grade was determined as per the level of differentiation—low (well differentiation), intermediate (moderate differentiation), and high (poor differentiation or undifferentiation). END was considered if at least 10 lymph nodes were sampled, provided that no positive nodes were identified. Time to surgery (TTS) referred to the duration between the date of cancer confirmation and the date of surgery.

Primary outcome variables were 5-year disease specific survival (DSS) and overall survival (OS). Time of DSS was calculated from the date of surgery to the date of cancer caused death or last follow-up, and time of OS was calculated from the date of surgery to the date of death or last follow-up.

### Statistic analysis

Missing data pattern in histologic grade was deemed not missing completely at random, its missing rate was 20.0%. Missing data was imputed using multiple imputation using Fully Conditional Specifications implemented by the multiple imputation by chained equations algorithm.

The patients were categorized into two groups, namely those who underwent END and those who did not. The Chi-square test was utilized to compare clinicopathological variables between the groups, and significant factors were identified for inclusion in propensity score-matching (PSM) with a ratio of 1:1. The impact of END on DSS and OS was assessed through univariate and multivariable analyses, and the results were presented using hazard ratios (HR) and a 95% confidence interval (CI). Statistical analyses were carried out using R 3.4.4, and a p-value of less than 0.05 was considered to be indicative of significance.

## Results

### Baseline date

A total of 180 patients were included in this study, with a mean age of 67 ± 10 years. Among them, 104 were male, and 76 were female. The majority of the patients were of white ethnicity. Most of the patients resided in urban areas and had a household income of less than $75,000. Approximately half of the study population (about 50%) were married. Curative surgery was performed in 129 patients within 1 month after diagnosis. The histologic grade of tumors was categorized as low in 38 patients, intermediate in 83 patients, and high in 59 patients. Among the study cohort, 74 patients received adjuvant radiotherapy, while 47 patients underwent adjuvant chemotherapy. When comparing the non-END group to the END group, it was found that patients who underwent END were more likely to be married (p = 0.007) and to receive radiation therapy (p < 0.001) (Table 1).

**Table 1:** Clinicopathologic variables between elective neck dissection (END) and non-END groups.

Variable	Non-END (n = 140)	END (n = 40)	p
Age			
< 65	44	16	
65+	96	24	0.310
Sex			
Male	79	25	
Female	61	15	0.493
Race			
White	98	32	
Others	42	8	0.213
Marital status			
Married	54	25	
Others	86	15	0.007
Area			
Rural	65	16	
Urban	75	24	0.471
TTS (month)			
≤ 1	101	28	
> 1	39	12	0.791
Income ($)			
< 75,000	76	26	
75,000+	64	14	0.228
Grade			
Low	33	5	
Intermediate	62	21	
High	45	14	0.311
Radiotherapy			
No	92	14	
Yes	48	26	< 0.001
Chemotherapy			
No	105	28	
Yes	35	12	0.525

*TTS* time to surgery.

### Survival analysis

During a median follow-up period of 25 months (range 0–225 months), 102 patients expired, of which 58 cases were attributable to cancer.

In univariate analysis, factors of race, marital status, and radiotherapy were associated with both DSS and OS, age older than 65 years (p = 0.009) predicted poorer OS, and END (p = 0.028) linked with better DSS. These variables were further incorporated in multivariate analysis. Other factors did not impact either DSS or OS (Table 2).

**Table 2:** Univariate analysis of predictors for disease specific survival (DSS) and overall survival (OS).

Variable	DSS	OS
Age (65 + vs < 65)	0.075	0.009
Sex (male vs female)	0.908	0.212
Race (white vs others)	0.006	0.011
Marital status (married vs others)	0.023	0.010
Area (urban vs rural)	0.072	0.127
TTS (> 1 month vs ≤ 1 month)	0.268	0.313
Income (75,000+$ vs < 75,000 $)	0.841	0.916
Grade (high vs intermediate vs low)	0.131	0.174
Radiotherapy (yes vs no)	0.001	< 0.001
Chemotherapy (yes vs no)	0.299	0.498
Neck management (END vs non-END)	0.028	0.170

*TTS* time to surgery, *END* elective neck dissection.

In the multivariate analysis, END did not offer any additional protective benefit as compared to the non-END group, with an HR of 0.79 [0.36–1.69] (p = 0.524). Other factors such as radiotherapy (p = 0.004, HR 0.41, 95% CI 0.22–0.76; p = 0.001, HR 0.44, 95% CI 0.28–0.70) and white race (p = 0.010, HR 0.49, 95% CI 0.28–0.84; p = 0.010, HR 0.57, 95% CI 0.37–0.88) emerged as independent predictors of both DSS and OS. Compared to other marital statuses, being married significantly predicted better OS (p = 0.041, HR 0.64, 95% CI 0.42–0.98) rather than DSS (p = 0.127, HR 0.64, 95% CI 0.36–1.13). Additionally, patients above the age of 65 years had a 0.7-fold increased risk of death as compared to those below 65 years of age (Table 3).

**Table 3:** Multivariate analysis of predictors for disease specific survival (DSS) and overall survival (OS).

Variable	DSS		OS	
	p	HR [95% CI]	p	HR [95% CI]
Age				
< 65				Ref
65+			0.030	1.69 [1.05–2.71]
Race				
Others		Ref		Ref
White	0.010	0.49 [0.28–0.84]	0.010	0.57 [0.37–0.88]
Marital				
Others				Ref
Married	0.127	0.64 [0.36–1.13]	0.041	0.64 [0.42–0.98]
Radiotherapy				
No		Ref		Ref
Yes	0.004	0.41 [0.22–0.76]	0.001	0.44 [0.28–0.70]
Neck management				
Non-EN D		Ref		
END	0.524	0.79 [0.36–1.69]		

### PSM

**Figure 2:**
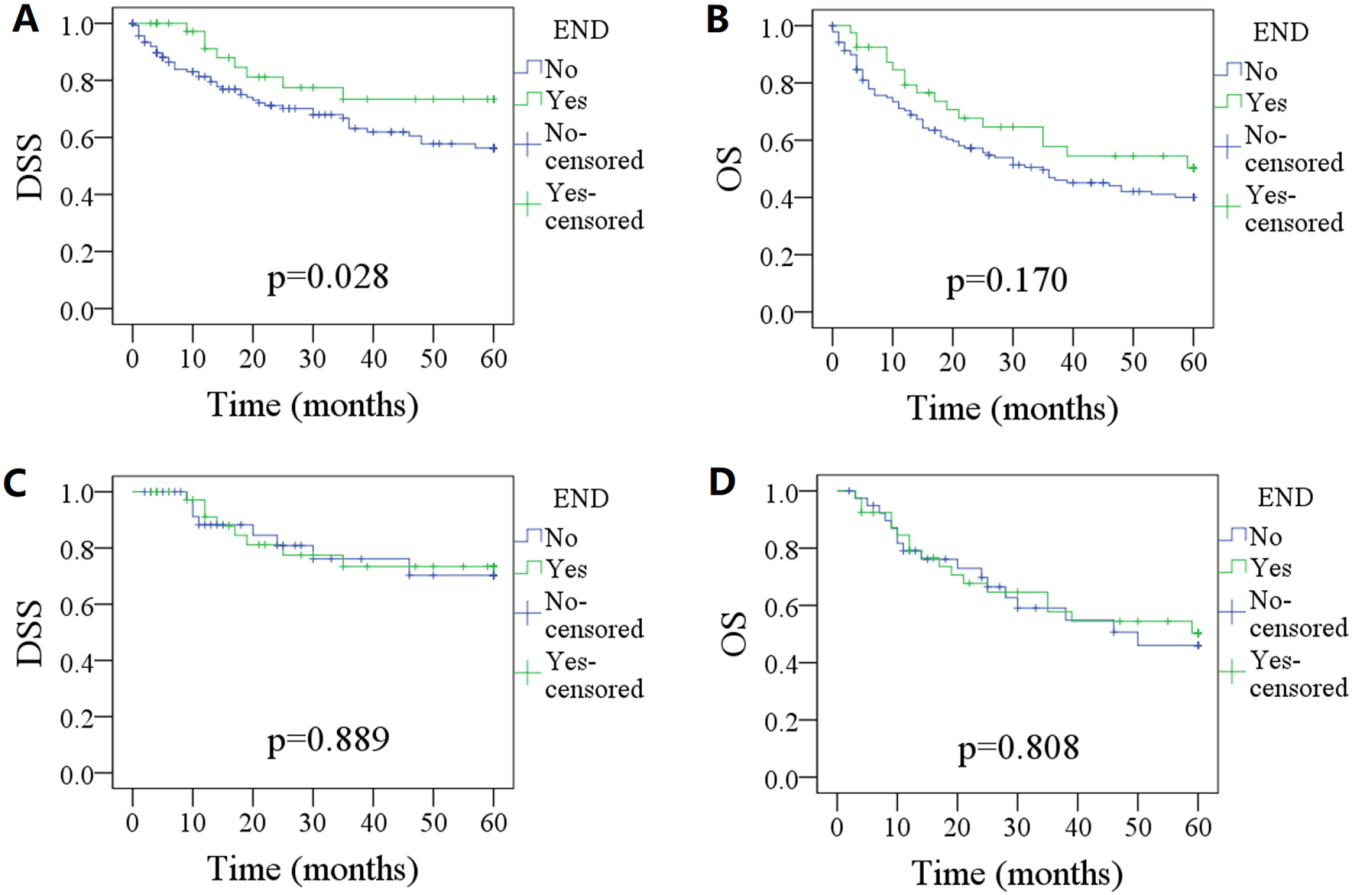
Comparison of disease specific survival (DSS) and overall survival (OS) between patients treated by elective neck dissection (END) and non-END before (**A**,**B**) and after (**C**,**D**) PSM.

Marital status and radiotherapy was calculated into PSM analysis with a ratio of 1:1, a total of 80 patients (40 from each group) were analyzed. Compared with non-END group, END was not associated with improved DSS (p = 0.889) or OS (p = 0.808) (Fig. 2).

### Occult metastasis

Among the END group, 9 patients (22.5%) had occult metastasis. Further analysis revealed that white patients had a rate of 12.5%, which was significantly lower than the rate of 62.5% observed in other races (p = 0.008). Additionally, while none of the patients with low grade disease developed occult metastasis, 50% of patients with high grade tumors developed it, the difference was statistically significant (p = 0.012) (Table 4).

**Table 4:** Predictors for occult metastasis in cT2 maxillary sinus squamous cell carcinoma.

Variable	Occult metastasis		p
	No (n = 31)	Yes (n = 9)	
Age			
< 65	10	6	
65+	21	3	0.120
Sex			
Male	21	4	
Female	10	5	0.255
Race			
White	28	4	
Others	3	5	0.008
Marital status			
Married	20	5	
Others	11	4	0.705
Area			
Rural	11	5	
Urban	20	4	0.441
TTS (month)			
≤ 1	21	7	
> 1	10	2	0.697
Income ($)			
< 75,000	19	7	
75,000+	12	2	0.453
Grade			
Low	5	0	
Intermediate	19	2	
High	7	7	0.012

In a further multivariable analysis, compared to patients of other races, white patients tended to have a decreased risk of occult metastasis, with a HR of 0.13 (95% CI 0.02–1.08). While low and intermediate grades had a comparable impact on occult metastasis (p = 0.126, HR 1.13, 95% CI 0.74–1.49), but high grade tumors were independently associated with an increased risk of occult metastasis (p = 0.011, HR 1.52, 95% CI 1.17–2.00) (Table 5).

**Table 5:** Multivariate analysis of predictors for occult metastasis in cT2 maxillary sinus squamous cell carcinoma.

Variable	p	OR [95%CI]
Race		
Others		Ref
White	0.059	0.13 [0.02–1.08]
Grade		
Low		Ref
Intermediate	0.126	1.13 [0.74–1.49]
High	0.011	1.52 [1.17–2.00]

## Discussion

Our study's most significant discovery was that cT2 MS-SCC was exceedingly uncommon, but it had a surprisingly high rate of occult metastasis (22.5%). This finding challenged our previously held beliefs and underscored the importance of careful surveillance in these patients. Additionally, we found that occult metastasis rate was independently associated with the tumor's histologic grade. Interestingly, after PSM analysis, END had no impact on either DSS or overall survival OS.

The occurrence of cT2 MS-SCC was extremely rare, representing less than 10% of all cases of MS-SCC. Hence, it was usually challenging to analyze this particular disease type within a single institution. Our study was the first to address this issue using a comprehensive public database. The presence of palate invasion in cT2 MS-SCC held significant clinical importance. Firstly, MS-SCC typically exhibited limited lymphatic drainage. However, invasion of the palate provided additional pathways for nodal metastasis. For instance, Cantù et al.^13^ reported the occurrence of LN involvement in a significant number of cases among 704 consecutive patients with paranasal sinus cancer, with T2 MS-SCC showing the highest rate. Ahn et al.^11^ investigated cancer cases of the nasal cavity or maxillary sinus and found that nodal involvement occurred in 182 out of 1283 eligible patients. The rates of occult metastasis were reported as 8.2% for T1 MS-SCC, 18.6% for T2 MS-SCC, 19.6% for T3 MS-SCC, and 23.4% for T4 MS-SCC. Similarly, Dubal et al.^10^ enrolled 854 cases of MS-SCC and observed neck involvement in 7.6% of T1 tumors, 22.2% of T2 tumors, 18.5% of T3 tumors, and 12.2% of T4 tumors. These findings highlighted that occult metastasis in cT2 MS-SCC was comparable to that observed in more advanced cT3/4 tumors. However, it was important to note that the existing evidence predominantly focuses on analyzing outcomes in advanced-stage MS-SCC^14^. Secondly, accurately calculating occult metastasis rates based on follow-up data was challenging due to the potential eradication of minimal metastatic foci by previous irradiation. In our study, we demonstrated an occult metastasis rate of 22.5% in cT2 MS-SCC by analyzing the samples obtained through END, which aligned with prior findings in this field.

The evaluation of predictors for occult metastasis had been extensively investigated in head and neck squamous cell carcinoma^15^. However, our study was the first to specifically explore potential factors for predicting LN involvement in MS-SCC. Our analysis revealed that histologic grade was the only factor that showed a significant association with LN metastasis. This finding was consistent with previous knowledge, as high-grade tumors were often characterized by a higher likelihood of LN involvement^16^. Ranasinghe et al.^17^ noted black patients were more likely than white patients to present with nodal metastasis, it was also confirmed by our analysis.

Our study findings, combined with prior evidence, confirmed that the rate of occult metastasis in cT2 MS-SCC was approximately 20%. This aligned with the principle that END should be recommended if the risk of occult metastasis exceeded 15–20%. However, it was worth noting that the question of whether END offered additional survival benefits in patients with cT2 MS-SCC had not been analyzed before. Janik et al. analyzed data from 47 patients with cancer of the nasal cavity and ethmoid sinus and found that although the results were not significant, patients treated by END tended to have fewer regional and distant metastases^18^. In a larger retrospective analysis of 927 MS-SCC cases, Sangal et al. reported that END reduced the 5-year hazard of death independently. However, further analysis showed that the significant finding existed only in T3 tumors, not T1/2 or T4 tumors^7^. Nevertheless, it was worth noting that the grouping of T1 and T2 stages into a single variable was not appropriate since they had significantly different occult metastasis rates. Bahig et al. evaluated the role of END in MS-SCC prognosis at the M. D. Anderson Cancer Center and surprisingly observed that the performance of END predicted worse progression-free survival and OS. It was suggested that this outcome may have been due to the inclusion of all histologic types in the study, with END being more likely to be performed on undifferentiated cancers rather than adenoid cystic carcinoma or mucoepidermoid cancer^3^. More recently, a SEER study extracted data from 777 N0M0 MS-SCC patients and demonstrated that END decreased the risk of cancer-caused death and overall death by approximately 30%. Notably, the protective effect appeared to be more apparent in patients who underwent both radiotherapy and END^6^. Indeed, while the SEER study showed the potential benefits of END in MS-SCC patients, it did not perform a subgroup analysis to clarify the impact of END in each tumor stage, including cT2 MS-SCC.

Our research found that patients who underwent END tended to be married and receive more adjuvant radiotherapy. This observation could be explained by several factors. Firstly, the detection and removal of LN metastases by END might had facilitated more effective control of the disease by radiation therapy. Additionally, it was possible that patients who tended to receive more intensive treatment, such as those who are married and had access to more social support, might had been more likely to undergo END or receive adjuvant radiotherapy. To account for these confounding factors, a PSM analysis was conducted in our research to minimize the potential impact of these variables on our results, it was noted that END was not associated with improved either DSS or OS.

The findings of our study were particularly fascinating. As previous studies had suggested, END could provide better prognoses for head and neck cancer patients with an occult metastasis risk exceeding 15–20%^19^. However, in the context of cT2 MS-SCC, this benefit was not confirmed in our study. It was possible that the local recurrence of cT2 MS-SCC accounted for the majority of treatment failure patterns, leaving little chance for a salvaged operation^20^. This recurrence pattern was evident in our study population, where the median survival time for patients who succumbed to the disease was only 15 months. Another potential explanation was that our patients were relatively older, and about half of the population died during follow-up. This suggested that many patients died before regional metastasis could become clinically detectable or increase the occult metastasis risk to the level where the benefits of END would become more apparent. In the treatment of cT2 MS-SCC, the resection of the palate often resulted in oral and nasal communication, as well as a decrease in speech and chewing function. However, END could provide proper recipient vessels for flap reconstruction, which might help to mitigate these adverse effects and improve treatment outcomes for patients.

Limitation in current study must be acknowledged, first, there was inherent bias within retrospective study, second, information of margin status was unknown, third, external validation was required before clinical application.

To summarize, cT2 MS-SCC was a rare condition that carried an occult metastasis rate of 22.5%, which was primarily influenced by histologic grade. END did not provide a significant survival benefit in terms of DSS or OS, it might still had a role to play in the management of this condition.

## Data Availability

All data generated or analyzed during this study are included in this published article. And the primary data could be achieved from the corresponding author.
